# Dentoskeletal effects of Class II malocclusion treatment with the Twin Block appliance in a Brazilian sample: A prospective study

**DOI:** 10.1590/2176-9451.19.1.036-045.oar

**Published:** 2014

**Authors:** Luciano Zilio Saikoski, Rodrigo Hermont Cançado, Fabrício Pinelli Valarelli, Karina Maria Salvatore de Freitas

**Affiliations:** 1 MSc in Orthodontics, Ingá College (UNINGÁ).; 2 Adjunct professor, Department of Orthodontics, postgraduate program, (UNINGÁ).; 3 Adjunct professor, Department of Orthodontics, Ingá College (UNINGÁ).

**Keywords:** Angle Class II malocclusion, Skull circumference, Functional orthodontic appliances, Prospective studies, Treatment outcome

## Abstract

**Objective:**

The aim of this study was to assess the dentoskeletal effects of Class II malocclusion treatment performed with the Twin Block appliance.

**Methods:**

The experimental group comprised 20 individuals with initial mean age of 11.76 years and was treated for a period of 1.13 years. The control group comprised 25 individuals with initial mean age of 11.39 years and a follow-up period of 1.07 years. Lateral cephalograms were taken at treatment onset and completion to assess treatment outcomes. Intergroup comparison was performed by means of the chi-square and independent t tests.

**Results:**

The Twin Block appliance did not show significant effects on the maxillary component. The mandibular component showed a statistically significant increase in the effective mandibular length (Co-Gn) and significant improvement in the maxillomandibular relationship. The maxillary and mandibular dentoalveolar components presented a significant inclination of anterior teeth in both arches. The maxillary incisors were lingually tipped and retruded, while the mandibular incisors were labially tipped and protruded.

**Conclusions:**

The Twin Block appliance has great effectiveness for correction of skeletal Class II malocclusion in individuals with growth potential. Most changes are of dentoalveolar nature with a large component of tooth inclination associated with a significant skeletal effect on the mandible.

## Abstract (pt)

**Objetivo:**

avaliar os efeitos dentoesqueléticos do tratamento da má oclusão de Classe II com o aparelho Twin Block comparado a um grupo controle.

**Métodos:**

o grupo experimental foi composto por 20 pacientes com idade inicial média de 11,76 anos e que foram tratados por um período de 1,13 anos. O grupo controle foi composto por 25 indivíduos com idade inicial média de 11,39 anos e que foram acompanhados por um período de 1,07 anos. Telerradiografias em norma lateral foram obtidas ao início e final do tratamento para avaliar as alterações decorrentes do tratamento. A comparação intergrupos foi realizada por meio do teste qui-quadrado e do teste *t* independente.

**Resultados:**

o aparelho Twin Block não apresentou alterações significativas no componente maxilar. O componente mandibular revelou um aumento estatisticamente significativo do crescimento efetivo da mandíbula (Co-Gn) e uma melhora significativa da relação existente entre as bases ósseas. Os componentes dentoalveolar superior e inferior apresentaram um significativo componente de inclinação dos dentes anteriores em ambas as arcadas. Os incisivos superiores foram inclinados para lingual e retruídos, ao passo que os incisivos inferiores foram inclinados para vestibular e protruídos.

**Conclusões:**

o aparelho Twin Block apresenta grande efetividade na correção da má oclusão de Classe II esquelética em pacientes em fase de crescimento. A maior parte das alterações ocorridas é de natureza dentoalveolar, com um grande componente de inclinação dentária associado a um significativo efeito esquelético na mandíbula.

## INTRODUCTION

Functional appliances have been widely used for treatment of skeletal Class II malocclusion. Even though a few clinicians do not recognize the great effectiveness of these appliances, scientific evidence about the fact that these appliances promote changes in jaw growth remains undefined.^1,2^

Some authors believe that there is little evidence to support the fact that functional appliances significantly alter mandibular growth.^3,4^ Conversely, other authors suggest that these appliances may have a significant influence over mandibular growth, when used in proper timing.^5,6,7^

The main changes caused by functional appliances are of dentoalveolar nature, including distalization of the maxillary posterior segment, lingual inclination of maxillary incisors, mesialization of the mandibular posterior segment and buccal inclination of mandibular incisors.^8^ The main vertical changes comprise restriction of vertical development of maxillary molars and stimulation of vertical development of mandibular molars.^8^

However, most of the aforementioned results have been obtained from retrospective studies, and a relatively small number of studies which aimed at assessing dentoskeletal changes were considered as prospective.^9-12^ Thus, this study prospectively assessed the dentoskeletal effects of the Twin Block appliance for treatment of the Class II malocclusion.

## MATERIAL AND METHODS

### Sample

This study was approved by the Institutional Review Board of Ingá College and all subjects in the sample signed an informed consent form before treatment onset. Sample size calculation was performed to determine the minimum number of individuals in each group. It was calculated considering α = 5% (type I error), β = 20% (type II error), estimated variability (s) of 1.5^13^ and a minimum difference of 2 mm to be detected (d) between the control and experimental groups. The results revealed a sample of 17 individuals in each group (accounting for occasional losses), with a test power of 80%. A sample of 19 individuals in each group allows a test power of 85%.

The prospective sample comprised 20 dental casts obtained at treatment onset (T_1_) and 40 lateral cephalograms obtained at onset (T_1_) and completion (T_2_) of orthopedic treatment of 20 individuals with Class II division 1 malocclusion. Twenty-five dental casts and 50 lateral cephalograms obtained from 25 individuals with Class II division 1 malocclusion, who did not receive treatment, comprised the control group. The cephalograms and dental casts in the control group were obtained from the files of the Department of Orthodontics of School of Dentistry - University of São Paulo/Bauru.

The experimental group comprised 20 individuals, 11 males and 9 females, with initial mean age of 11.76 ± 1.64 years presenting Class II division 1 malocclusion at treatment onset and who were treated with the modified Twin Block functional orthopedic appliance. The mean treatment time was 1.13 ± 0.40 years and the final mean age was 12.89 ± 1.56 years. With regard to the initial severity of anteroposterior relationship between the permanent first molars assessed on the dental casts, 9 individuals presented full Class II, 3 presented 3/4 of Class II, 7 presented 1/2 Class II and 1 presented 1/4 of Class II.

The control group comprised 25 untreated individuals, 14 males and 11 females, with Class II division 1 malocclusion, with initial mean age of 11.39 ± 1.35 years. The mean follow-up time was 1.07 ± 0.17 years and the final mean age was 12.46 ± 1.38 years. As for the initial severity of anteroposterior relationship between the permanent first molars assessed on the dental casts, 4 individuals presented full Class II, 6 presented 3/4 of Class II, 9 presented 1/2 Class II and 6 presented 1/4 of Class II.

The inclusion criteria for the experimental group were: 1) presence of Class II division 1 malocclusion assessed on the dental casts and clinically confirmed (no cephalometric criterion was used to determine that individuals presented skeletal Class II with ANB values greater than 4 degrees); 2) crowding in the mandibular arch not greater than 4 mm; 3) no previous orthodontic treatment; 4) presence of clinically observable facial convexity.

#### Description of the modified Twin-Block appliance

**Figure 1:**
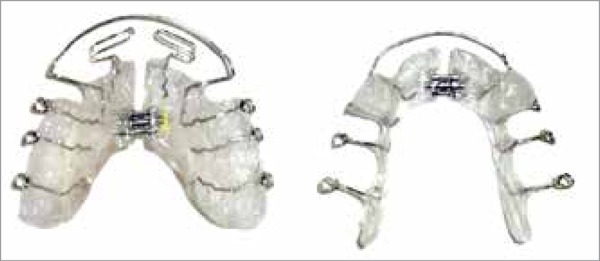
Modified Twin Block appliance.

**Figure 2:**
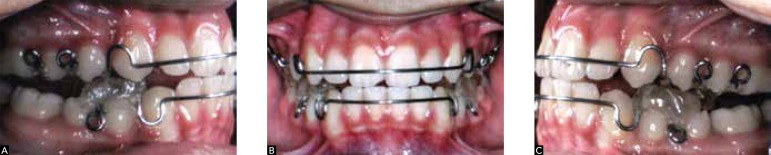
Twin Block appliance in use - A) Right lateral view. B) Frontal view. C) Left lateral view.

Maxillary portion - composed of an acrylic base covering the hard palate, open at the midpalatal suture line with a Dentaurum^®^ 6.5 mm expanding screw, allowing transverse expansion of the maxillary arch. It contains an anterior Hawley bow used to enhance retention, retract the lip musculature and control the inclinations of maxillary incisors. The appliance has simple coils on the palatal region of maxillary central and lateral incisors for tongue pressure control and teeth uprighting. The appliance retention is achieved in posterior teeth with Benac clasps, which allow activation and present good flexibility due to the great amount of wire employed for fabrication. The acrylic blocks are placed on the occlusal surface of posterior teeth with enough height to allow disocclusion of anterior teeth. The anterior portion of planes present an angle of 70 degrees, which, in combination with the mandibular planes, keeps the mandible protruded (Figs 1 and 2).

The mandibular portion is composed of an acrylic base on the lingual alveolar ridge, with anterior Hawley bow to control the inclination of incisors. The presence of a Dentaurum^®^ 5.5-mm expanding screw on the midline allows correction of small lingual inclinations of posterior teeth. Benac clasps are used for appliance retention on the posterior portion, and, if the bow is not sufficient in the anterior portion, an acrylic coverage should be applied on the edges of mandibular incisors. The planes are located ahead, at the region of the first premolars, and are extended up to the canines in order to achieve greater strength. They are fabricated at 70 degrees to fit with the maxillary portion of the appliance, keeping the mandible in a more anterior position. Plane height is compatible with the upper plane, without contact with teeth in the maxillary arch (Figs 1 and 2). The individuals were instructed to use the modified Twin Block for an approximate period of 20h/day.

#### Lateral cephalograms

Aiming to verify the dentoskeletal changes of the modified Twin Block appliance, lateral cephalograms obtained at treatment onset and completion were assessed and compared to the control group. All radiographic images were obtained with the lips at rest and in maximum intercuspation, with the aid of the Broadbent cephalostat to standardize head positioning. All cephalograms in the sample were performed in three difference machines and the magnification of each appliance was determined in order to allow greater accuracy of results. The different machines presented distinct magnification percentages which ranged from 6% to 10.94%.

#### Cephalometric tracing and achievement of measurements

The cephalograms were digitized at a resolution of 9600 x 4800 dpi in a Microtek ScanMaker i800 scanner (Microtek International, Inc., Carson, CA, USA) connected to a Pentium microcomputer. The images were transferred to the Dolphin Imaging Premium 10.5 software (Dolphin Imaging & Management Solutions, Chatsworth, CA, USA) through which the cephalometric points of interest were marked and measurements involving the planes and lines were obtained.

#### Cephalometric measurements employed (Figs 3, 4, 5 and 6)

**Figure 3:**
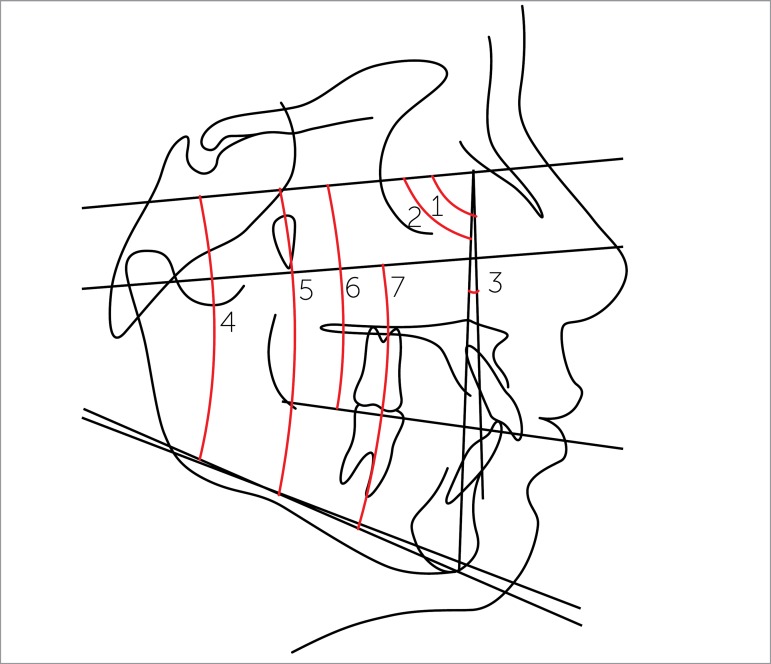
Skeletal angular cephalometric measurements: 1) SNA; 2) SNB; 3) ANB; 4) SN.GoMe; 5) SN.GoGn; 6) SN.Ocl; 7) FMA.

**Figure 4:**
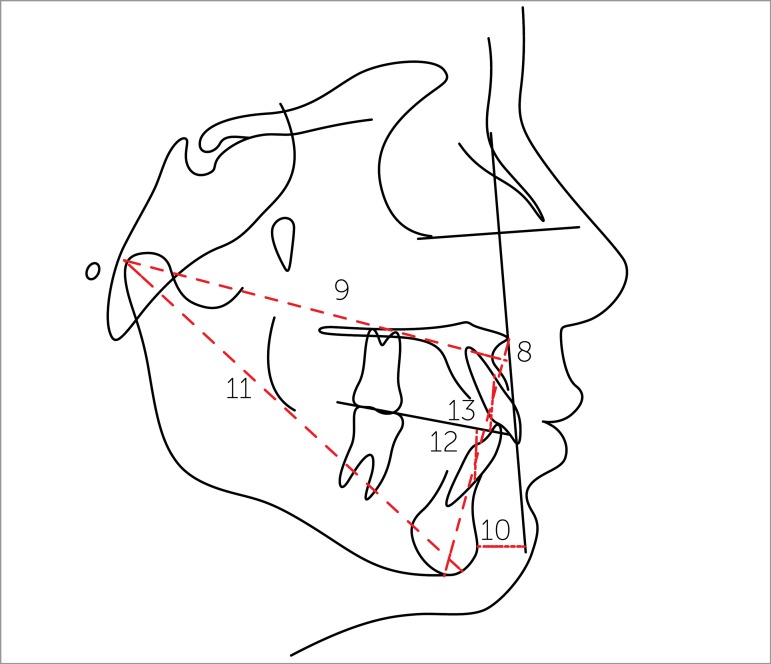
Linear skeletal cephalometric measurements: 8) A-Nperp; 9) Co-A; 10) P-Nperp; 11) Co-Gn ;12) Wits; 13) LAFH.

**Figure 5:**
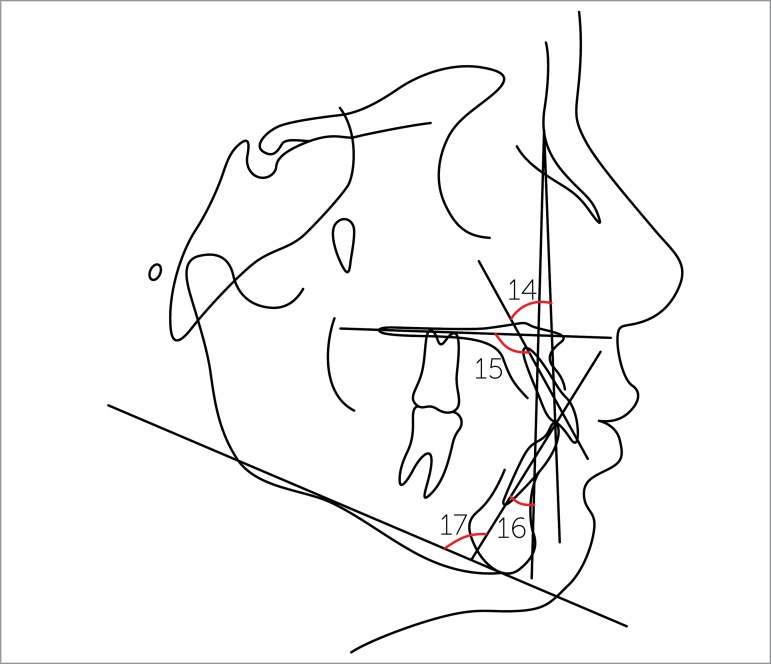
Angular dental cephalometric measurements: 14) 1.NA; 15) 1.PP; 16) 1.NB; 17) IMPA.

**Figure 6:**
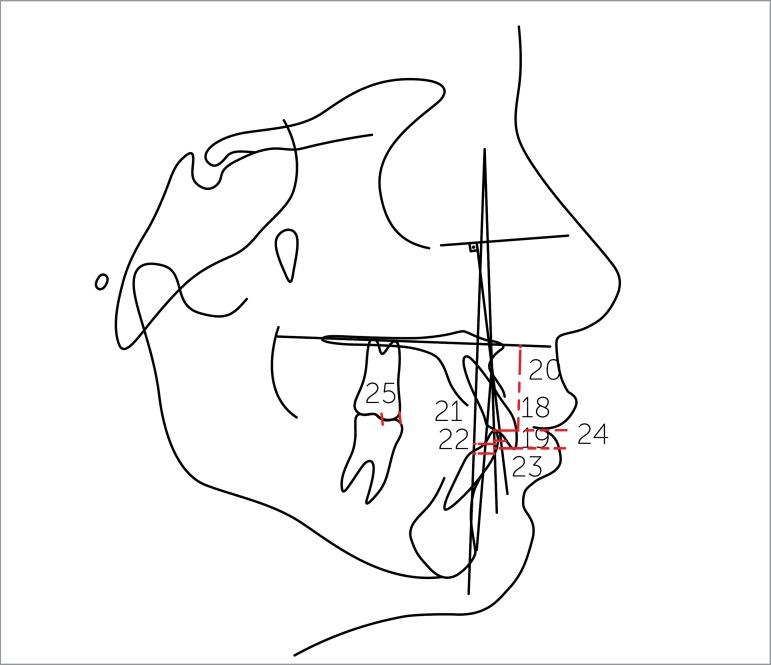
Linear dental cephalometric measurements: 18) 1-NA; 19) 1-Aperp; 20) 1-PP; 21) 1-NB; 22) 1-AP; 23) overjet; 24) overbite; 25) molar relationship.

The following cephalometric measurements were used in this study:

1. Maxillary component: SNA, A-Nperp and Co-A.
2. Mandibular component: SNB, P-Nperp and Co-Gn.
3. Maxillomandibular relationship: ANB and Wits.
4. Growth pattern: SN.GoGn, SN.GoMe, SN.Ocl, FMA and LAFH.
5. Maxillary dentoalveolar component: 1.NA, 1-NA, 1-Aperp, 1.PP and 1-PP.
6. Mandibular dentoalveolar component: 1.NB, 1-NB, 1-AP and IMPA.
7. Dental relationships: overjet, overbite and molar relationship.

## STATISTICAL ANALYSIS

### Method error

To evaluate the intra-examiner error, all measurements were repeated by the same investigator on 30 lateral cephalograms randomly selected after a three-week interval. Application of the mathematical formula proposed by Dahlberg (Se^2^ = Σd^2^/2n) allowed estimation of casual errors.^14^ Systematic errors were assessed by the dependent t test.^15,16^

### Intergroup comparison

The Kolmogorov-Smirnov test was applied to analyze if cephalometric data in the experimental and control groups presented normal distribution. The results revealed that the cephalometric variables presented normal distribution in both groups and in all periods analyzed (P > 0.05). Thus, parametric tests were used for intergroup comparison. The compatibility between experimental and control groups in relation to the initial (T_1_) and final mean ages (T_2_) and the treatment/follow-up time was assessed by the independent t test. The chi-square test was used to verify the compatibility between groups with regard to gender distribution and anteroposterior severity existing between molars.

The independent t test was used for intergroup comparison at the initial (T_1_) and final periods (T_2_) and to assess changes between the initial and final periods (T_2_-T_1_) in both groups. Bonferroni correction was used for false-positive control (type I error), and differences were considered statistically significant at P < (0.05/24) = 0.002.

All statistical tests were performed by means of the Statistica for Windows 7.0 software (Stat Soft Inc., Tulsa, Oklahoma, USA).

## RESULTS

Three variables (SNA, SN.GoGn and LAFH) presented systematic error (P < 0.05) and the amplitude of casual errors ranged from 0.32 (ANB) to 2.39 (LAFH).

The experimental and control groups were compatible in initial and final age, treatment/follow-up time, gender distribution and severity of anteroposterior relationship existing between molars (Tables 1, 2 and 3).

**Table 1:** Evaluation of compatibility between groups considering initial age, final age and treatment/follow-up time (independent t test).

Variables (years)	Experimental group (n = 20)	Control group (n = 25)	P
	Mean ± S.D.	Mean ± S.D.	
Initial age	11.76 ± 1.64	11.39 ± 1.35	0.4063
Final age	12.89 ± 1.56	12.45 ± 1.38	0.3239
Treatment/follow-up time	1.13 ± 0.40	1.07 ± 0.17	0.4773

**Table 2:** Comparison of sex distribution in the two groups (chi-square test).

Group	Sex		Total
	Female	Male	
Experimental	9	11	20
Control	11	14	25
Total	20	25	45
χ^2^= 0.005; df = 1; P = 0.9465			

**Table 3:** Result of the chi-square test for comparison between experimental and control groups with regard to the severity of existing anteroposterior molar relationship.

Severity	Experimental group(n = 20)	Control group(n = 25)
¼ Class II	1	6
½ Class II	7	9
¾ Class II	3	6
Full Class II	9	4
χ^2^= 6.2663; df = 3; P = 0.0993		

At treatment onset (T_1_), the experimental and control groups presented moderate cephalometric compatibility, with the variables ANB and Wits in the maxillomandibular relationship component presenting the worst relationship between jaws in the experimental group (P < 0.002). In the maxillary dentoalveolar component, the 1-Aperp variable revealed that maxillary incisors in the experimental group were significantly more buccally inclined and protruded in the maxilla (P < 0.002). As for the dental relationship component, the overjet variable significantly increased in relation to the control group (P < 0.002) (Table 4).

**Table 4:** Results of the independent t test for comparison between experimental and control groups at the initial period (T1).

Variables	Experimental Group (T1) (n = 20)	Control Group (T1) (n = 25)	p
	Mean ± S.D.	Mean ± S.D.	
**Maxillary component**			
SNA (degrees)	84.51 ± 3.51	83.30 ± 3.16	0.2270
A-Nperp (mm)	0.78 ± 2.98	-0.18 ± 2.70	0.2618
Co-A (mm)	85.45 ± 3.38	83.67 ± 4.80	0.1686
**Mandibular component**			
SNB (degrees)	77.33 ± 4.10	78.59 ± 3.49	0.2723
P-Nperp (mm)	-8.95 ± 6.64	-6.30 ± 4.83	0.1287
Co-Gn (mm)	108.49 ± 6.60	108.14 ± 6.27	0.8570
**Maxillomandibular relationship**			
ANB (degrees)	7.19 ± 2.27	4.69 ± 1.66	**0.0001**
Wits (mm)	3.84 ± 2.65	0.50 ± 2.34	0.0001
**Growth pattern**			
SN.GoGn (degrees)	30.46 ± 5.24	29.88 ± 4.95	0.7049
SN.Ocl (degrees)	13.00 ± 5.20	14.57 ± 2.99	0.2095
FMA (degrees)	26.58 ± 4.85	25.83 ± 4.06	0.5743
LAFH (mm)	61.16 ± 4.03	58.49 ± 4.55	0.0461
**Maxillary dentoalveolar component**			
1.NA (degrees)	29.48 ± 6.75	24.73 ± 6.29	0.0190
1-NA (mm)	5.03 ± 2.10	3.44 ± 1.87	0.0107
1-Aperp (mm)	6.23 ± 1.74	4.40 ± 1.05	**0.0001**
1.PP (degrees)	120.68 ± 5.63	115.20 ± 5.80	0.0027
1-PP (mm)	26.35 ± 1.86	25.37 ± 2.76	0.1837
**Mandibular dentoalveolar component**			
1-NB (mm)	5.14 ± 2.44	3.92 ± 1.97	0.0713
1.NB (degrees)	26.18 ± 6.98	24.40 ± 6.34	0.3760
1-AP (mm)	-0.15 ± 2.12	0.37 ± 2.16	0.4213
IMPA (degrees)	95.92 ± 8.16	93.47 ± 6.59	0.2715
**Dental relationships**			
Overjet (mm)	9.16 ± 2.10	5.61 ± 2.61	**0.0000**
Overbite (mm)	4.59 ± 2.50	3.29 ± 1.73	0.0464
Molar relationship (mm)	1.90 ± 1.54	0.40 ± 1.55	0.0024

At treatment completion (T_2_), the growth pattern, evaluated by the LAFH variable, was significantly greater in the experimental group in comparison to the control group. In the mandibular dentoalveolar component, the experimental group presented significantly more protruded and buccally inclined mandibular incisors in comparison to the control group. In the evaluation of dental relationships, the experimental group presented significantly smaller molar relationship in comparison to the control group (Table 5).

**Table 5:** Results of the independent t test for comparison between experimental and control groups at the final period (T2).

Variables	Experimental Group (T2) (n = 20)	Control Group (T2) (n = 25)	p
	Mean ± S.D.	Mean ± S.D.	
**Maxillary component**			
SNA (degrees)	84.18 ± 4.55	82.99 ± 3.26	0.3117
A-Nperp (mm)	0.50 ± 3.36	-0.48 ± 2.51	0.2728
Co-A (mm)	87.26 ± 3.68	84.84 ± 4.39	0.0562
**Mandibular component**			
SNB (degrees)	78.61 ± 4.66	78.84 ± 3.90	0.8523
P-Nperp (mm)	-7.08 ± 7.14	-5.93 ± 4.81	0.5256
Co-Gn (mm)	115.00 ± 6.72	110.48 ± 6.21	0.0242
**Maxillomandibular relationship**			
ANB (degrees)	5.59 ± 1.83	4.14 ± 1.69	0.0087
Wits (mm)	1.67 ± 3.15	0.16 ± 3.02	0.1083
**Growth pattern**			
SN.GoGn (degrees)	29.94 ± 5.39	28.96 ± 4.79	0.5223
SN.Ocl (degrees)	14.37 ± 5.13	13.70 ± 3.02	0.5823
FMA (degrees)	25.91 ± 4.58	25.26 ± 3.40	0.5885
LAFH (mm)	64.34 ± 4.00	59.02 ± 4.43	**0.0014**
**Maxillary dentoalveolar component**			
1.NA (degrees)	22.42 ± 5.11	25.13 ± 3.92	0.0502
1-NA (mm)	3.25 ± 1.78	4.04 ± 1.61	0.1265
1-Aperp (mm)	4.78 ± 1.52	4.88 ± 1.22	0.8001
1.PP (degrees)	113.68 ± 4.38	114.96 ± 4.19	0.3221
1-PP (mm)	27.84 ± 2.09	26.02 ± 2.67	0.0167
**Mandibular dentoalveolar component**			
1-NB (mm)	7.10 ± 2.27	4.10 ± 1.74	**0.0000**
1.NB (degrees)	32.35 ± 6.28	25.25 ± 5.10	**0.0001**
1-AP (mm)	2.85 ± 2.10	0.74 ± 2.00	**0.0013**
IMPA (degrees)	101.43 ± 6.95	94.68 ± 5.02	**0.0005**
**Dental relationships**			
Overjet (mm)	3.88 ± 1.46	5.43 ± 2.09	0.0071
Overbite (mm)	3.04 ± 1.44	3.30 ± 1.53	0.5633
Molar relationship (mm)	-1.87 ± 2.24	0.42 ± 1.22	**0.0001**

Comparison of dentoskeletal changes (T_2_-T_1_) between the experimental and control groups revealed that, in relation to the mandibular component, the experimental group exhibited a significantly greater increase in mandibular length (Co-Gn). As for the growth pattern component, the Sn.Ocl variable exhibited significantly greater increase in the experimental group in comparison to the control group. With regard to the maxillary dentoalveolar component, the experimental group presented greater and significant lingual inclination and retrusion of maxillary incisors in comparison to the control group. In the mandibular dentoalveolar component, the experimental group exhibited greater and significant buccal inclination and protrusion of mandibular incisors in comparison to the control group. In the analysis of dental relationships, the experimental group exhibited significantly greater reduction in overjet and molar relationship when compared to the control group (Table 6).

**Table 6:** Results of the independent t test for comparison of changes (T2-T1) between experimental and control groups.

Variables	Experimental Group (T2) (n = 20)	Control Group (T2) (n = 25)	p
	Mean ± S.D.	Mean ± S.D.	
**Maxillary component**			
SNA (degrees)	-0.33 ± 1.69	-0.30 ± 1.47	0.9564
A-Nperp (mm)	-0.29 ± 2.46	-0.29 ± 1.90	0.9915
Co-A (mm)	1.80 ± 2.60	1.17 ± 1.23	0.2870
**Mandibular component**			
SNB (degrees)	1.27 ± 1.41	0.26 ± 1.29	0.0152
P-Nperp (mm)	1.88 ± 4.35	0.37 ± 2.82	0.1674
Co-Gn (mm)	6.51 ± 3.13	2.34 ± 1.56	**0.0000**
**Maxillomandibular relationship**			
ANB (degrees)	-1.60 ± 1.55	-0.54 ± 1.10	0.0108
Wits (mm)	-2.16 ± 2.78	-0.34 ± 2.30	0.0210
**Growth pattern**			
SN.GoGn (degrees)	-0.53 ± 1.67	-0.92 ± 2.09	0.4911
SN.Ocl (degrees)	1.37 ± 2.38	-0.87 ± 1.87	**0.0010**
FMA (degrees)	-0.68 ± 2.21	-0.57 ± 2.57	0.8877
LAFH (mm)	3.18 ± 3.12	1.49 ± 1.44	0.0201
**Maxillary dentoalveolar component**			
1.NA (degrees)	-7.06 ± 6.11	0.40 ± 4.38	**0.0000**
1-NA (mm)	-1.77 ± 1.62	0.60 ± 1.45	**0.0000**
1-Aperp (mm)	-1.44 ± 1.33	0.49 ± 1.16	**0.0000**
1.PP (degrees)	-7.00 ± 6.41	-0.24 ± 3.93	**0.0001**
1-PP (mm)	1.50 ± 1.64	0.65 ± 1.27	0.0583
**Mandibular dentoalveolar component**			
1-NB (mm)	1.96 ± 1.83	0.18 ± 0.95	**0.0001**
1.NB (degrees)	6.17 ± 5.96	0.85 ± 3.29	**0.0004**
1-AP (mm)	3.00 ± 1.97	0.36 ± 1.10	**0.0000**
IMPA (degrees)	5.51 ± 6.33	1.20 ± 3.53	0.0060
**Dental relationships**			
Overjet (mm)	-5.29 ± 2.20	-0.18 ± 1.28	**0.0000**
Overbite (mm)	-1.55 ± 2.73	0.01 ± 1.39	0.0169
Molar relationship (mm)	-3.76 ± 2.32	0.02 ± 1.39	**0.0000**

## DISCUSSION

The use of removable functional orthopedic appliances in growing individuals with skeletal Class II has demonstrated to have some advantages promoted by treatment of Class II malocclusion in two stages (functional orthopedics and fixed appliance).^11,17^ Reduction in overjet at early ages, better relationship between the jaws, reduction in facial convexity and shorter treatment time with fixed appliances are factors that encourage treatment of Class II malocclusion in two stages.^9^

Conversely, some authors have demonstrated that treatment of Class II malocclusion performed in one stage in the permanent dentition (fixed appliance) is more efficient in comparison to treatment performed in two stages, given that similar occlusal results are obtained in significantly shorter treatment time.^18,19,20^

Investigations into the actual dentoskeletal changes obtained with the Twin Block appliance in the first treatment stage did not reveal any restriction of anterior maxillary displacement (Table 6). This result suggests that treatment of Class II malocclusion with the Twin Block did not present any significant extraoral effect, as reported in previous studies.^17,21^

Evaluation of the mandibular component revealed a statistically significant increase of 4.17 mm in the mandibular length (Co-Gn) with anterior displacement of the Gonion, two changes that are desirable in the treatment of individuals with skeletal Class II malocclusion (Table 6). It was not possible to determine if the increase in the Co-Gn variable was caused by an increase in mandibular length or mandibular repositioning. Some authors have also evidenced similar changes in relation to mandibular length.^9,11,17,22^ However, the functional orthopedic appliances promote a greater increase in mandibular length within shorter treatment time, yet the final mandibular length at completion of the growth period is not significantly greater in comparison to untreated individuals. This characteristic of functional appliances is known in the literature as the mortgage of mandibular growth.^2,23^ Improvement in mandibular retrognathism was also observed in individuals in the experimental group, who presented a greater increase in the SNB variable (1.01 degrees) when compared to the control group (Table 6). This change probably contributed to reduce facial convexity in individuals in the experimental group.

A probable lingual movement of the roots of mandibular incisors may promote alveolar remodeling, changing the position of point B to a more posterior position and, as a consequence, reducing the SNB variable. The mandibular incisors presented significant buccal inclination and protrusion, yet evidenced an increase in the SNB angle (Table 6). Previous studies also found similar changes in the evaluation of cephalometric effects promoted by the use of functional appliances.^11,21^

Evaluation of the maxillomandibular relationship component revealed that mandibular growth and/or repositioning did not promote significant changes in ANB and Wits variables with consequent reduction in skeletal discrepancy between jaws in individuals in the experimental group (Table 6). This result does not agree with previous studies, since several studies in the literature demonstrate the great effectiveness of functional appliances in achieving a better relationship between maxilla and mandible.^17,24,25^

With regard to growth pattern, there was a non-significant increase in LAFH (1.69 mm) in individuals in the experimental group compared to the control group, with consequent clockwise rotation of the occlusal plane, as observed by the significant increase in the SN.Ocl variable (Table 6). These effects were probably caused by selective wear of the acrylic in contact with the mandibular posterior teeth, allowing greater vertical development of these teeth, which contributes for correction of Class II relationship, curve of Spee and deep bite in the individuals.^26,27,28^

The maxillary and mandibular dentoalveolar components presented a significant component of inclination of anterior teeth in both arches. The maxillary incisors were lingually inclined and retruded, while the mandibular incisors were buccally inclined and protruded (Table 6). These dentoalveolar changes significantly contributed for correction of the overjet.^9,17,22^ However, excessive inclination of incisors should be carefully controlled, since they substantially reduce the potential of changes of orthopedic nature.^9^

In the evaluation of dental relationships, there was a significant reduction of 5.11 mm in the overjet and of 3.78 mm in molar relationship in comparison to the control group. These changes contribute to correct the anteroposterior discrepancy in individuals with Class II malocclusion (Table 6). These results represent a desirable consequence of treatment of skeletal Class II malocclusion, and are established by the combination of dentoalveolar and skeletal changes that occurred in the experimental group.^29,30^

## CONCLUSION

Based on the methods applied and the results achieved, it is reasonable to conclude that the Twin Block appliance presented great effectiveness for correction of Class II malocclusion in growing individuals. Most changes were of dentoalveolar nature with a marked component of dental inclination associated with a significant skeletal effect on the mandible.
